# Modified craniotomy procedures and virtual reality technology for accurate localization of the frontal branch of MMA in revascularization surgery

**DOI:** 10.1038/s41598-024-65123-z

**Published:** 2024-06-22

**Authors:** Liu Chuang-Hong, Xu Hong, Kong Gang

**Affiliations:** 1 Neurosurgery Department, Changshu First People’s Hospital Affiliated to Soochow University, Soochow City, 215500 China; 2 Neurosurgery Department, First People’s Hospital of Changshu City, Changshu, Soochow China

**Keywords:** Moyamoya disease, Cerebral revascularization, Carotid artery, Internal, Virtual reality, Imaging, Three-dimensional, Cerebrovascular disorders, Brain, 3-D reconstruction

## Abstract

The frontal branch of middle meningeal artery (MMA) can easily be damaged during revascularization surgery. To precise locate it and minimize its injury, we propose a set of modified craniotomy procedures combined with simple virtual reality (VR) technology based on three-dimensional (3D) Slicer simply, economically, and efficiently. Patients with Moyamoya disease (MMD) and internal carotid artery occlusion (ICAO) who received revascularization from January 2015 to December 2022 were divided into two groups based on the methods used to locate the MMA: traditional methods and precise MMA locating with VR technology. Patient demographics and clinical characteristics were analyzed to compare the preservation rates of MMA. The distances between this artery and bony anatomical landmarks were also measured to better understand its localization. There was no significant difference in baseline characteristics between the two groups. The precise MMA locating group exhibited a significantly higher preservation rate of the frontal branch of MMA (p = 0.037, 91.7% vs. 68.2%). Over 77% of patients had their frontal branch of MMA partially or completely surrounded by bony structures to varying degrees. Therefore, the combination of modified craniotomy procedures, 3D Slicer, and simple VR technology represents an economical, efficient, and operationally simple strategy.

## Introduction

Surgical revascularization is widely recognized as an effective intervention for patients with MMD and ICAO, as it improves cerebral hemodynamics and prevents subsequent ischemic and hemorrhagic strokes. There are three main categories of surgical revascularization techniques: direct, indirect, and a combination of direct and indirect techniques. In most cases, the MMA plays a crucial role due to the following reasons: (1) MMA serves as the donor artery in some indirect anastomosis procedures, such as encephalo-duro-myo-synangiosis (EDMS)^1^, encephalo-duro-arterio-synangiosis (EDAS)^2^ and encephalo-duro-arterio-myosynangiosis (EDAMS)^3^; (2) the collateral circulation of the MMA and cortical branches of anterior cerebral artery (ACA) or middle cerebral artery (MCA) may develop spontaneously^1,4–6^. Especially, its frontal branch provides collateral blood flow to ACA territory through the falx^7,8^; (3) Rare but important, Ophthalmic artery can directly originate from MMA in approximately 1.5% cases^9^.

Proper MMA protection is of great importance for favorable outcomes in patients with MMD and ICAO. However, this task can be difficult to accomplish due to the complex course and anatomical variation of the MMA. It has been reported that 49–75% of patients exhibit a bony duct structure, through which the frontal branch of the MMA passes, completely or incompletely^10,11^. Patients with such variation are at a higher risk of sustaining injury during frontotemporal craniotomy. Several studies have focused on the relationship between the MMA and pterion to assist in achieving accurate MMA localization. For example, the study conducted by Siyan Ma et al. proposed the pterion as a consistent reference point for the MMA, but this approach only produced positive results in two-thirds of cases without providing guidance on how to avoid the damage of MMA^11^. In contrast, other studies have proposed measures to protect the MMA, including: (1) Satoshi Hori's et al.^12^ suggested the drilling holes on the skull in areas traversed by the MMA and performing a heart-shaped flap to protect MMA. (2) Meanwhile, Xu Bin et al.^1^ utilized personalized designed bone flaps to safeguard the main trunks of the MMA. However, these methods may not be effective due to variations in the shape and location of the pterion, which do not consistently correlate with the frontal branch of MMA. Otherwise, none of them mentioned specific technique for precisely locating the MMA. In addition, there have been studies attempting to use Indocyanine green (ICG) imaging^13^ or surgical navigation systems^14^ to locate the MMA. Both can accurately locate the MMA, but each has its drawbacks: due to the obstruction of the skull, the former can only clearly display and locate the MMA in 37% of patients, while the latter requires expensive navigation equipment. Therefore, there is an urgent clinical need for an economical, convenient, and universally applicable method for locating the MMA.

3D Slicer is a free and open-source software package for medical image analysis and visualization. It was originally developed at Brigham and Women's Hospital in Boston, Massachusetts in the late 1990s^15^. Since then, it has evolved into a widely used platform for quantitative medical image analysis, with applications ranging from neuroimaging to radiation therapy planning^16^.

Precise identification and localization of the frontal branch of MMA is essential for clinical outcomes. To that end, we present a solution that entails the combination of a modified craniotomy procedure and 3D Slicer (version 4.10.1, Boston MA, USA) based virtual reality technology. This approach enables accurate identification and localization of the frontal branch of MMA, thereby facilitating individualized bone flap formation and reducing the incidence of MMA injury. Our method is both economical and user-friendly, as it requires no additional radiological examination and has produced positive results in our study.

## Methods

### Patients

A prospective database of patient information was maintained, and clinical data were reviewed retrospectively from medical records, radiographic reports, and office visit records. A total of 71 patients who underwent revascularization at Changshu First People's Hospital, Soochow University between January 2015 and December 2022 were included in the study. Inclusion criteria: (1) Patients diagnosed with MMD or ICAO by preoperative cerebral digital subtraction angiography (DSA), whose clinical symptoms and computer tomography perfusion (CTP) or magnetic resonance perfusion (MRP) evaluation suggest the need for cerebral vascular reconstruction surgery; (2) EDMS with or without superficial temporal artery- MCA (STA-MCA) bypass performed by the first author; (3) A follow-up period of 3–6 months after surgery, including cerebral DSA to evaluate MMA and collateral formation.

Two groups were established based on the method for locating the MMA: traditional craniotomy group (early group, 2015–2020) and precise MMA locating group (latter group, 2020–2022). The former group employed bone window images of non-contrast computer tomography (NCCT) scan to roughly determine the frontal branch of MMA, followed by drilling based on the surgeon's expertise and creating an individually designed bone window. In contrast, the latter group employed a simple VR technology using 3D slicer and a mobile phone app to accurately locate the MMA, followed by precise drilling.

### Identification and location of MMA and craniotomy procedures in early surgical groups

In this group, two methods were used for the identification and location of MMA: (1) using the STA as a landmark by first confirming the parietal branch through arterial pulsation or ultrasound, and then referring to the lateral DSA image of the external carotid artery to locate the MMA. The frontal branch of MMA is typically located 2–3 cm anterior to the parietal branch of STA and parallel to it; (2) using the coronal suture as a landmark by recognizing its position relationship with the groove of the MMA on skull X-rays.

**Figure 1:**
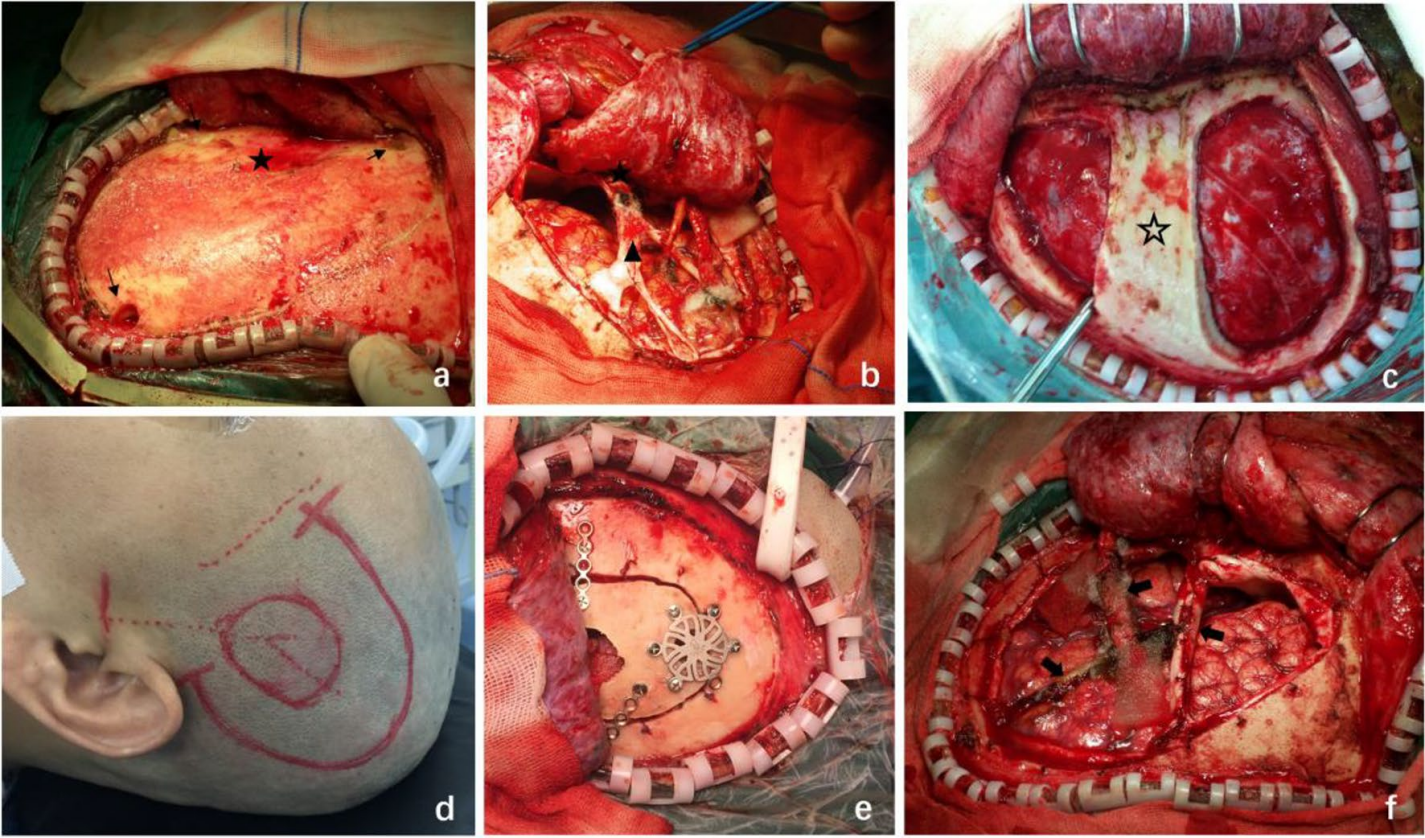
A set of modified standard craniotomy procedures used for maximizing the preservation of MMA integrity. (**a**) Three burr holes (small arrow) were drilled on the skull to form a heart-shaped bone flap, exposing only the distal MMA without exposing the bony canal or deep groove where the proximal MMA is located (Black pentagram). If necessary, the fourth and or the fifth burr would be placed at the black pentagram. (**b**) A ridge (Black pentagram) preserved at the lower edge of the bone window without damaging the bony canal and the MMA within. The dura mater (Triangle) was then flipped over and sutured. (**c**) The “bone bridge method” was used to avoid damaging the MMA and existed anastomosis in a patient who had bony canal type MMA. Bone flaps were formed before and after the bone bridge (Hollow pentagram) for STA-MCA bypass. (**d,e**) The “small bone window” was formed, which is just large enough for STA-MCA anastomosis to avoid damaging the MMA and anastomosis in a patient with deep grove type MMA. (**f**) For patients with minor MMA surface damage and poor hemostasis effect under compression, the "Chinese spring roll suture method" (Solid Arrow) can effectively stop bleeding and preserve the integrity of the main trunk of the MMA.

The craniotomy procedure involved a frontotemporal craniotomy through 3–5 burr holes using a self-stop grinding drill. The frontal and temporal burr holes were placed in the same manner as in a traditional craniotomy, and additional burr holes were used around the pterion as needed. For cases without spontaneous collateral anastomosis formation between the cerebral cortex and MMA (collaterals from MMA), a routine frontal–temporal craniotomy was performed using the "bone ridge technique" for preservation of the bone ridge at the sphenoid bone's lesser wing to avoid unnecessary exposure of the proximal part of MMA and bony canal/deep grove, as depicted in Fig. 1a,b. To ensure the integrity of the MMA, various strategies were employed including: (1) radial cutting of the dura mater while retaining the trunk of the MMA by partially cutting the dura mater near the branch and flipping the dura mater strips (Fig. 1b). (2) The "bone bridge" method for patients with extensive collaterals from MMA, which created two bone windows functioning as a bridge over the MMA (Fig. 1c). Alternatively, the "small bone flap" method can also be used (Fig. 1d,e), which only forms a small bone window sufficient for direct bypass, avoiding unnecessary exposure of the meninges, the MMA and spontaneous anastomosis. (3) The 'Chinese spring rolls' strategy was employed to stop bleeding when temporal compression was insufficient (Fig. 1f).

### Identification and location of MMA and craniotomy procedures in precise MMA locating group

**Figure 2:**
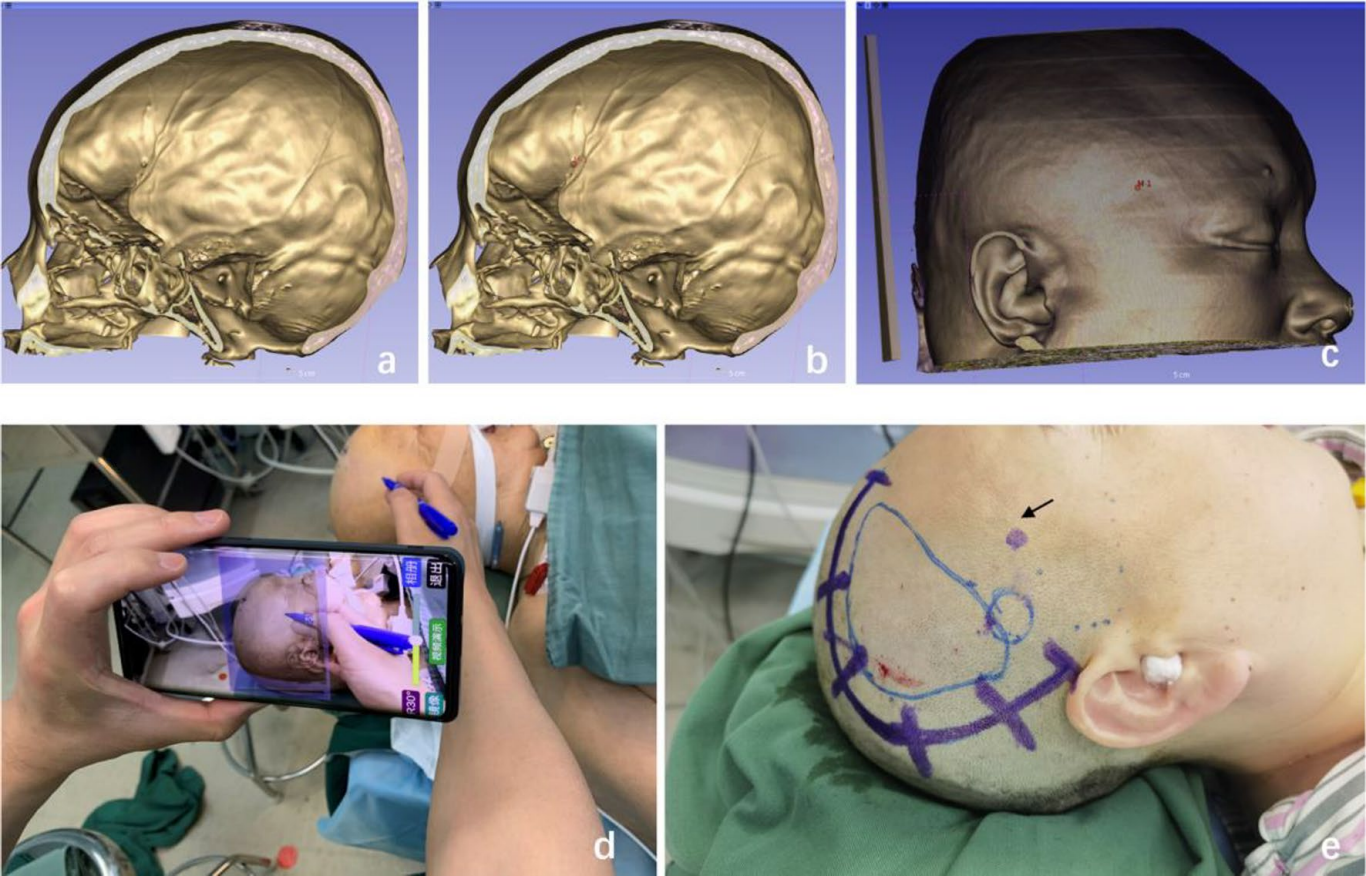
The process of accurately locating the MMA using simple virtual reality technology based on 3D Slicer. (**a**) 3D skull reconstruction by 3D Slicer revealed that the patient's right MMA is a bony canal type. (**b**) The bony canal endpoint is marked in red (M1). (**c**) Parameters and transparency are adjusted to display the scalp and point M1 simultaneously. (**d**,**e**) Save the image to a smart mobile and projected it onto the patient's scalp using a mobile app. The image angle, size, and transparency are adjusted until the virtual image completely overlaps with the patient's head, and the surface projection of the M1 point (arrow) is marked on the patient's scalp. The endpoint of the MMA bone canal is completely outside the Dangerous Rectangle, indicating that a heart-shaped bone flap is not necessary in this case. Instead, the MMA can be exposed by drilling one hole and directly milling the bone flap.

To efficiently locate MMA without increasing radiation exposure or patient costs, we innovatively combined 3D Slicer software (version 4.10.1, Boston MA, USA) with a mobile app (Digital Brain, The Meda Intelligent Medical Doctoral Team, https://pan.baidu.com/s/1y2A0180p2IjZl4unpERYzA?pwd=1111) and simple VR technology. The process involved the following steps: (1) The patient's NCCT images were imported into the 3D Slicer software for 3D skull reconstruction using the volume rendering module (Fig. 2a), preferably with CT angiography data if available. (2) Analyze the types of middle meningeal vascular grooves along with the relationship between the MMA and bony structure. (3) Then, A key marker was added at the endpoint of the bony canal or deep groove (Fig. 2b), followed by adjustment of imaging parameters to display the scalp clearly and adjust transparency to reveal the key marker and scalp simultaneously. Alternatively, the 3D scalp module and key marker image were exported separately and superimposed using image synthesis software (Fig. 2c). (4) Next, the saved image was projected onto the patient's scalp using an APP called Digital Brain, and the position of the key marker was marked (Fig. 2d,e). (5) Using the key marker as a guide, bone tissue should be carefully removed with a high-speed grinding drill if the endpoint is within the range of the bone flap, preserving and protecting the MMA intact before employing the same craniotomy techniques as the first group. However, if the endpoint lies outside the bony window, a heart-shaped bone flap is not necessary, and a bone flap can be formed directly without concern for damaging the frontal branch of MMA.

### Classification of the frontal branch of MMA

Currently, there is no standardized classification system for the frontal branch of MMA. Ma et al. proposed a system based on the anterior branch's path near the sphenoid's lesser wing and adjacent parietal bone^11^. They categorized the branch as a groove or canal, which further classified as complete, incomplete, or disrupted. But frontal branch of MMA that not embraced in the bone canal or groove were not included in this study. In a separate study by Satoshi Hori^12^, the frontal branch of the MMA was classified into three types: the bridge, monorail, and tunnel types. The tunnel type corresponded to the complete canal type, and the monorail type corresponded to the incomplete canal type, while the bridge type was defined as the frontal branch of the MMA passing through a shallow groove resembling a bridge over a river.

**Figure 3:**
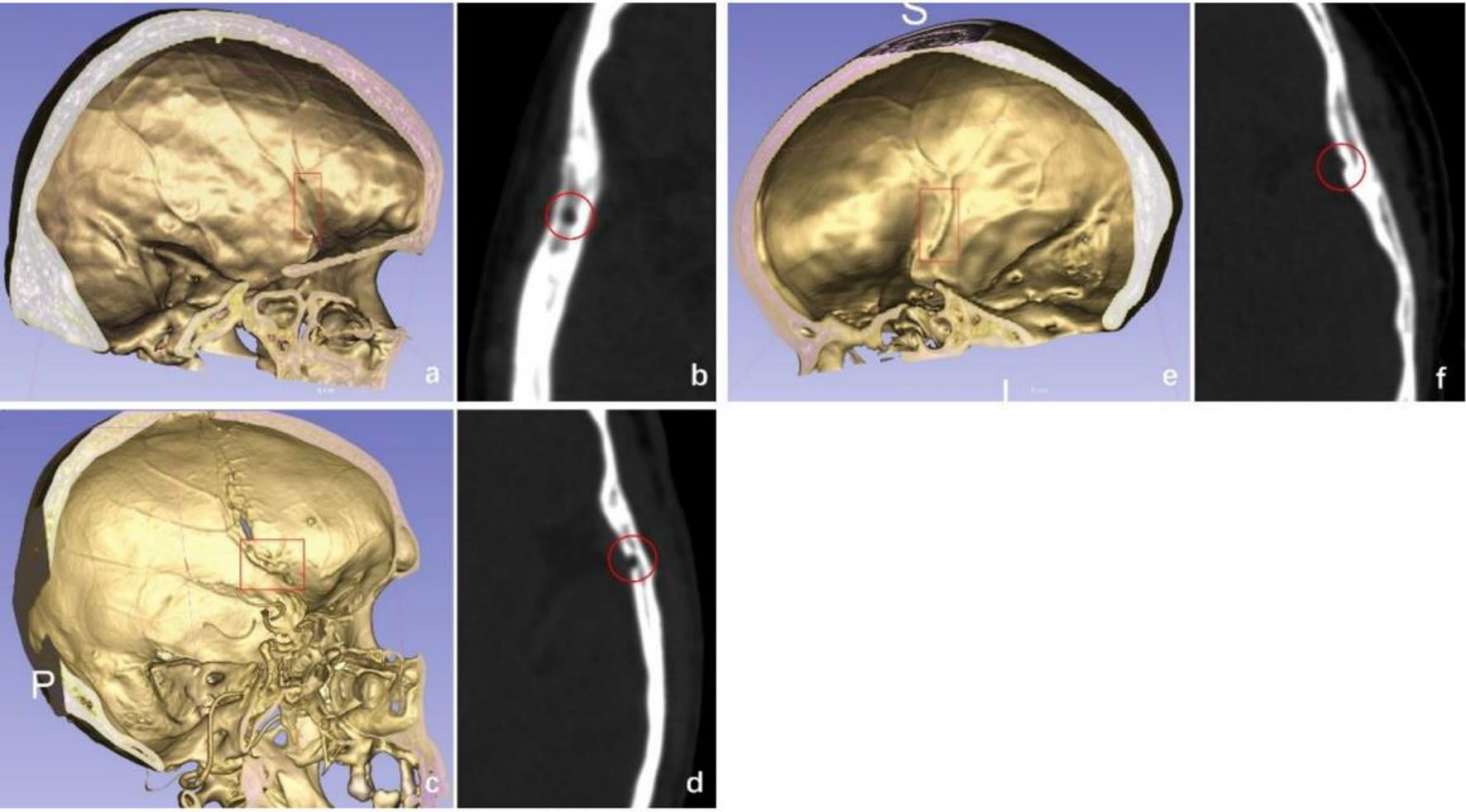
An exemplary illustration of the classification of the frontal branch of the MMA. (**a**,**b**) The bony canal type. (**c**,**d**) the deep groove type. (**e**,**f**) the bridge type.

Our study included a review of the literature and our experiences, and we classified the frontal branch of MMA into three types (Fig. 3): canal type (including both complete and disrupted canals as per Ma's classification), deep groove type (with a groove cross-section greater than 1/2 circle), and bridge type (with no bony groove or a groove cross-section less than 1/2 circle). This classification system was chosen for its simplicity, clarity, and relevance to preserving the MMA during surgery.

### Relations between frontal branch of MMA and bony structure

**Figure 4:**
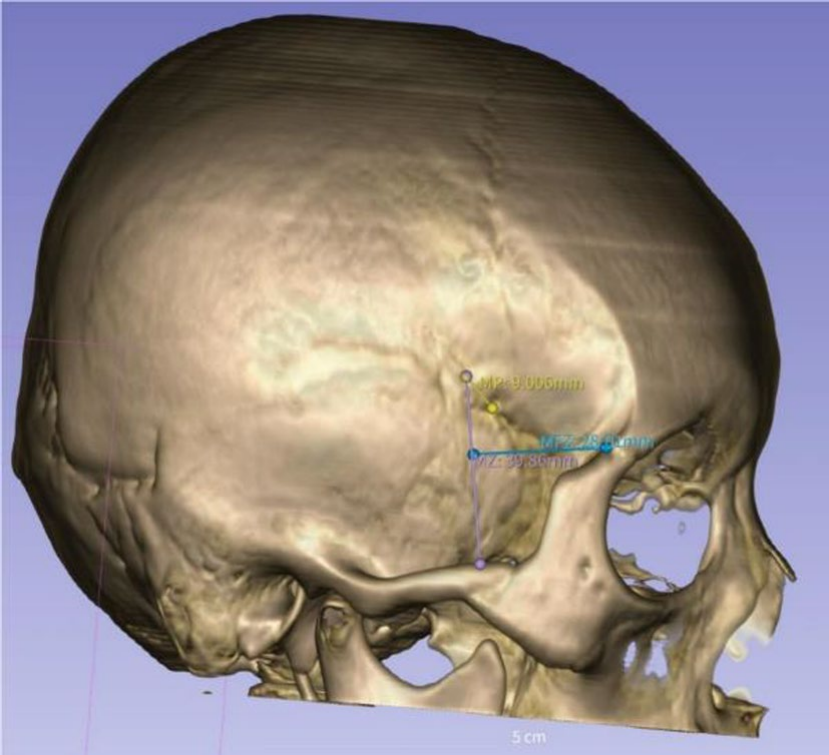
The measurements obtained in this study.

To achieve a more accurate surface marking of the frontal branch of MMA and evaluate its correlation with the bony structure, we conducted measurements on 3D skull models from both groups. The NCCT data of the first group of patients was imported into 3D Slicer for reconstruction and retrospective analysis, using the same method as the second group. The following measurements were obtained and analyzed (Fig. 4):

- the projection of the endpoint where the frontal branch of the MMA separates from the bony canal or groove was defined as the key point (M), and the distance from the center of pterion (P) to this point was defined as MP. For sphenoparietal and frontotemporal types of the pterion, the center was defined as the midpoint of a straight line connecting the ends of the suture. In other types of the pterion, the center was determined as the center of the smallest circle that contained the edge of all relevant bones.
- Defined the vertical distances from the key point to the superior border of the zygomatic arch as MZ.
- The horizontal distances from the key point to the posterolateral margin of the frontozygomatic suture was defined as MFZ. The line MFZ should be perpendicular to the line MZ.

### Outcome evaluation and data analyses

The focus was on comparing the preservation rates of MMA between the two groups and their influencing factors. In addition, to better understand the localization of the frontal branch of MMA, a secondary focus was on the distance between the frontal branch of MMA and bony anatomical landmarks. The preservation of MMA was assessed based on surgical records and cerebral DSA results obtained 3–6 months after the operation. 3D Slicer software was utilized for three-dimensional skull reconstruction and measurement of appropriate anatomical parameters.

Normality tests were conducted for numerical variables, followed by means and t-tests for normally distributed data and medians and non-parametric tests for non-normal data. Categorical variables were expressed in percentages and analyzed using χ2 tests or Fisher's exact probability method. A two-sided p-value of 0.05 was considered statistically significant. Data collection was performed using Microsoft Excel (version 16, Microsoft Corp.), and all statistical analysis and charting were carried out using IBM SPSS Statistic version 23.0.

### Ethics approval

Approval was obtained from the ethics committee of Changshu First People’s Hospital Affiliated to Soochow University. The procedures used in this study adhere to the tenets of the Declaration of Helsinki.

### Approval for human experiments

Informed consent was obtained from all subjects and/or their legal guardian(s) prior to their participation in the study. This includes obtaining specific consent to publish any information or images that could lead to the identification of a study participant in an online open-access publication.

## Results

### Clinical results

Out of the 71 patients, three were excluded due to incomplete imaging data. The traditional craniotomy procedure was utilized in 64.7% (44 cases), while 35.3% (24 cases) underwent a precisely located frontal branch of MMA. Both groups were comparable in baseline characteristics, as presented in Table 1.

**Table 1:** The demographic and clinical characteristics of the two patient groups.

	Traditional craniotomy group	Precise MMA locating group	Test value	p value
Sex				
Male	25	11	0.752*	0.451
Female	19	13		
Age (years)	46.09 ± 10.12	46.46 ± 14.91	− 0.121^#^	0.904
Diagnosis				
MMD	30	18	0.348*	0.592
ICAO	14	6		
Comorbidity				
DM	3	2	9.94**	1.000
HTN	15	7	0.172*	0.678
Collaterals from MMA pre-op	11	6	0.00*	1.000
Surgery method				
STA-MCA bypass only	4	1	0.921**	0.642
EDMS only	12	5		
STA-MCA bypass + EDMS	28	18		
GOS discharge	4.80 ± 0.85	4.96 ± 0.20	− 0.92^#^	0.361

*DM* diabetes mellitus, *HTN* hypertension, *GOS* Glasgow outcome scale.

*Pearson Chi-square test.

**Fisher's exact Chi-Square test.

^#^Independent samples t-Test.

### MMA preservation condition in this study

Compared with the traditional craniotomy group, the precise MMA locating group had a significantly higher preservation rate of the frontal branch of MMA on the surgical side (p = 0.037), which were 68.2% (30/44) and 91.7% (22/24), respectively. Further subgroup analysis based on the presence of collaterals from MMA before surgery revealed that there was no significant difference in the MMA preservation rate between the two groups when collaterals from MMA were present. However, when collaterals from MMA were absent, there was a significant difference in the MMA preservation rate between the two groups (Table 2). However, there were no statistically significant differences in the MMA frontal branch subtype, MP value, MZ value, MFZ value, and surgical method between the two groups (Table 3).

**Table 2:** Comparison of MMA preservation rates in patients with/without collaterals from MMA pre operation between the two groups.

	Traditional craniotomy group	Precise MMA locating group	Test value	p value
MMA preserved	30/44	22/24	4.760*	0.029
Collaterals from MMA pre-op				
Preserved	10	6	5.134**	0.054
Damaged	1	0		
No collaterals from MMA pre-op				
Preserved	20	16	4.488*	0.034
Damaged	13	2		

*Pearson Chi-square test.

**Fisher's exact Chi-square test.

**Table 3:** Comparison of MMA anatomical features and preservation rates between patients in the traditional craniotomy group and the MMA precise locating group.

	Traditional craniotomy group	Precise MMA locating group	Test value	p value
Op MMA type				
Canal type	17	8	1.975**	0.414
Deep grove type	20	9		
Bridge type	7	7		
Op MP (mm)	15.59 (10.08, 21.10)	15.53 (7.88, 19.94)	0.903^Δ^	0.367
Op MZ (mm)	48.64 ± 8.46	45.24 ± 8.22	1.596^#^	0.115
Op MFZ (mm)	32.61 ± 6.08	32.10 ± 5.94	0.330^#^	0.743
Op side				
Left side	25	8	3.429*	0.079
Right side	19	16		
Surgery method				
Bypass only	4	1	0.921**	0.640
EDMS only	12	5		
Bypass + EDMS	28	18		
Op preserved				
Preserved	30	22	4.76*	0.037
Not preserved	14	2		

*Pearson Chi-square test.

**Fisher's exact Chi-square test.

^#^Independent samples t-test.

^Δ^Mann-Whitney test.

### Relationship between the frontal branch of MMA and bony anatomical landmarks

**Figure 5:**
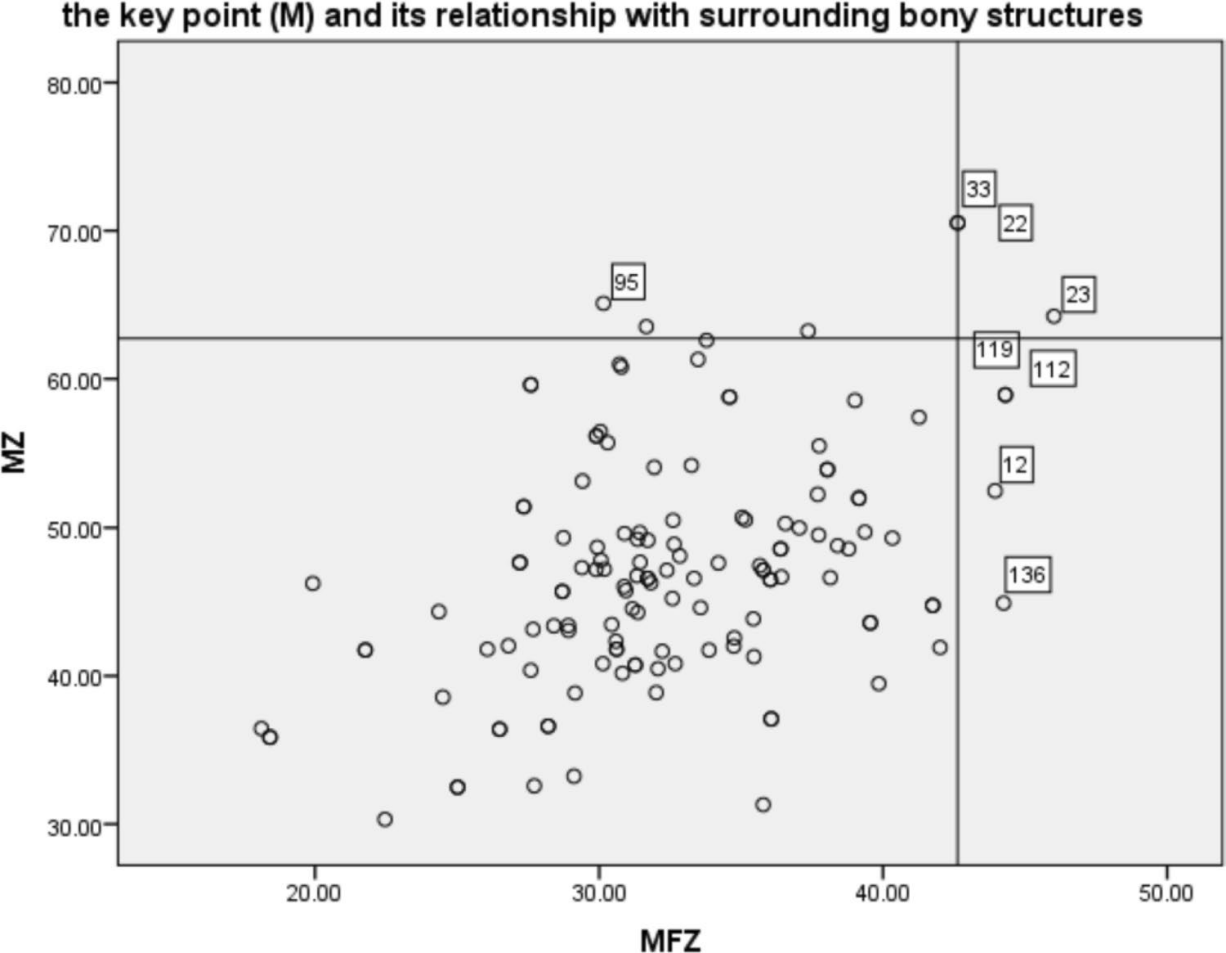
The key point (M) and its relationship with surrounding bony structures. Using the 95th percentile of MZ and MFZ as reference lines for the X and Y axes, respectively, it was found that in 94% of cases, the M point was located within the first quadrant in the lower left corner, with only 8 cases having the M point located outside this Dangerous Rectangle.

We conducted measurements on 3D skull models to accurately surface mark the frontal branch of MMA and assess its correlation with bony structures. Among the 68 patients, one patient who underwent left decompressive craniectomy due to intracranial hemorrhage at another hospital was excluded, resulting in 68 measurements for the right MMA and 67 measurements for the left MMA. Analysis revealed that over 77% of patients had their frontal branch of MMA partially or completely surrounded bony structures to varying degrees. The deep groove type was the most prevalent in the left frontal branch of MMA (44.8%), followed by the bony canal type (38.8%, 26/67). On the right side, the proportion of bony canal and deep groove types were comparable (36.8% and 35.3%, respectively). However, there was no significant statistical difference in the distribution of the three types on either side (p = 0.225). The distance between the key point M and the middle of pterion showed a large variation, with a median of 14.70 mm and a range from 2.82 to 37.75 mm. There was no statistically significant difference between the left and right sides. The variation in distance between the point M and the superior border of the zygomatic arch and the outer edge of the posterior lateral suture of the frontozygomatic junction was small. The average values of MZ and MFZ were 47.33 mm and 47.96 mm, respectively. There was no significant statistical difference between the left and right sides for the aforementioned measurements, as shown in Table 4. A scatter plot (Fig. 5) was generated using MZ and MF as the Y and X axes, respectively. The 95th percentile of both MZ and MFZ were used as reference lines, and it was found that 94% of them were located within the rectangular area formed by the two reference lines and the axes. These results suggest that it is safe to perform the bone flap in any of the other three quadrants outside of this rectangular area, thereby minimizing the risk of MMA injury.

**Table 4:** Classification of the frontal branch of the MMA and its relationship with surrounding bony structures.

	Total	Right side	Left side	Test value	p value
MMA type					
Canal type	51	25	26	2.980*	0.225
Deep grove type	54	24	30		
Bridge type	30	19	11		
MP (mm)	14.70 (8.89,19.24)	14.70 (8.63,19.09)	14.60 (9.30, 19.28)	− 0.048^Δ^	0.961
MZ (mm)	47.33 ± 7.96	48.05 ± 8.47	46.59 ± 7.41	− 1.069^#^	0.287
MFZ (mm)	32.57 ± 5.57	32.92 ± 5.53	32.22 ± 5.63	− 0.73^#^	0.467

*Pearson Chi-square test.

^#^Independent samples t-test.

^Δ^Mann–Whitney test.

## Discussion

MMD and ICAO are major risk factors for cerebral vascular disease, which often require surgical intervention. During surgery, the MMA is at risk of injury, which can result in serious neurological complications. Therefore, it is important to understand the importance of MMA in the surgical treatment of cerebral vascular disease and how to protect it during surgery.

Initially, people believed that the MMA was located within the middle meningeal groove. Subsequently, clinical and anatomical studies reveal that a majority of patients' MMA branches can pass through the bony canal or tunnel structure^10–12,17,18^. Discoveries by Shimizu et al. show that this tunnel is present in 75.6% of cases, with the majority being bilateral^10^. The length of the tunnel and the distance from the superior orbital fissure were 3–23 mm (mean: 12.2 mm) and 11–33 mm (mean: 18.9 mm), respectively. Depending on the strength of fixation in the tunnel, the patterns of MMA damage range from avulsion from the tunnel with tearing at the proximal or distal end, to removal of that portion of the vessel contained in the tunnel with tearing at the proximal and distal tunnel ends. Harthmann and colleagues^18^ measured some key parameters related to the MMA. Of note, they observed that the length of the bony tunnel is 13.6 ± 7.8 mm on the right side and 14.9 ± 7.3 mm on the left side. Honnegowda et al.^19^ replicated Harthmann's study and obtained similar results, with lengths of the left and right canals measuring 13.7 ± 9.3 mm and 12.8 ± 4.5 mm, respectively.

The above research helps us to better understand the anatomical structure of the MMA, but with certain limitations in clinical application: during the craniotomy, it is difficult to accurately determine the starting point, end point, and precise location of the bony canal on the surface of the skull. Research by Siyan Ma et al.^11^ highlights the practical value of surface anatomical features, revealing that a circular area with a one-centimeter diameter centered on the midpoint of the pterion intersects the anterior branch of the MMA in 68% of skulls; in the remaining skulls, the artery was located just a few millimeters posteriorly.

Our study aims to further this research by directly measuring the distance of MP, MZ and MFZ, all of which are easily measurable surface markers in clinical practice. Our study showed no significant statistical differences in the MP, MZ, and MFZ values between the left and right sides. As shown in Fig. 5, our findings show that only 6% of patients have the endpoint of the bony canal or deep groove outside of “Dangerous Rectangle”, making it safe to form bone flaps outside of this range.

To protect the MMA, there are three major types of techniques: (1) Completely avoid exposing its main trunk: the "small bone window", "bone bridge" method, and form the bone flap outside the range of “Dangerous Rectangle” mentioned above; (2) Only expose the distal portion of the MMA, without exposing the bony canal or deep groove, such as “Bone ridge technique"^1,12^; (3) Fully expose the MMA: drilling out of the lesser wing under a surgical microscope was essential to preserve the tunnel-type MMA^12,19,20^.

Among them, the first two are the safest and most effective; although the "drilling" method can improve the preservation rate of MMA, it is not always successful. Why? Our research data found that the maximum values of MP, MZ, and MFZ were 37.75 mm, 70.53 mm, and 46.01 mm, respectively. For cases with such long bony canal or deep grooves, the formation of bone flaps cannot be performed outside of “Dangerous Rectangle”. Is it possible for us to preserve the integrity of the MMA well?

Fujimoto et al. conducted a study in 2017 examining the morphology and histology of intracranial arteries in 50 dry skulls and 28 cadaveric heads^20^. The study revealed that most of the dry skull specimens had bony canals. An important discovery in this study is that the MMA within the bony canal was surrounded by collagen tissues. This histological finding has clinical significance as it enables safe exposure and preservation of the anatomical basis of MMA during surgical procedures.

The important prerequisite for using the above method is to accurately locate the MMA and its position in the bone canal and deep groove. To accurately locate the MMA, there is an urgent need for an easy, economical, and precise method, as current methods such as CT, magnetic resonance (MR), ICG angiography, and Doppler ultrasound have limitations, either not precise enough, or complex and costly.

Overall, the MMA preservation rate in the precise localization group was significantly higher than that in the traditional localization group (91.7% vs. 68%). Subsequently, we conducted subgroup analysis based on the presence or absence of collaterals from MMA before surgery.

It is worth noting that for patients with collaterals from MMA before surgery, the "bone bridge" method and "small bone flap" method were commonly used during craniotomy to avoid damaging the MMA by not exposing its trunk. Therefore, patients in this subgroup, regardless of whether they came from the traditional or precise localization group, had a high MMA preservation rate and showed no statistically significant difference (10/11, 90.91% vs. 6/6, 100%, p = 0.054). Only one patient from the traditional localization group suffered from MMA injury due to inaccurate MMA localization during bone flap formation.

For patients without collaterals from MMA before surgery, the "bone ridge" method or direct bone flap formation was often used during craniotomy. For this subgroup, accurate localization of the MMA can contribute to a higher MMA preservation rate theoretically, and our study has confirmed it. In this subgroup, patients from the precise localization group had a significantly higher MMA preservation rate compared to those from the traditional localization group (16/18, 88.89% vs. 20/33, 60.61%, p = 0.034). In the precise localization group, there were two cases of MMA injury, both of which were attributed to tight adhesion between the dura mater and the skull. Specifically, one patient had the MMA frontal branch directly milled off by the milling cutter during bone flap formation, while the other patient suffered from injury while detaching the bone flap and dura mater. In the traditional localization group, thirteen cases of MMA injury occurred, 6 of which resulted from inaccurate MMA localization, causing damage to the bony canal and groove during bone flap formation. Although five patients avoided injuring the bony canal and groove during bone flap formation, they directly milled off the MMA frontal branch due to tight adhesion between the dura mater and the skull. The remaining two patients suffered from injury while detaching the skull and dura mater.

To efficiently locate MMA without increasing radiation exposure or patient costs, we innovatively combined 3D Slicer software with a mobile app and simple VR technology. 3D Slicer is an open-source medical image processing software that initially developed by the National Institutes of Health in the United States, it has become one of the most popular software in the field of medical imaging. The following are the main features of 3D Slicer: (1) Support for multiple image data formats. (2) Advanced image processing techniques, such as image registration, segmentation, reconstruction, etc. (3) Extensible plugin system: 3D Slicer's plugin system is very flexible and can be extended. These plugins include various medical image processing algorithms, visualization tools, surgical navigation, etc. (4) Open source and free. All the features make our strategy efficient and free. This method only requires importation of NCCT data into 3D Slicer for rapid reconstruction, analysis, and image export to the mobile phone. The entire process can generally be completed in 10–20 min. Consequently, this strategy is a cost-effective, efficient, and straightforward methodology.

## Limitations

The study also has some limitations. For instance, the anatomical measurements were conducted solely by the first author using method of taking the average of three measurements, instead of using multiple observers. Therefore, it is unclear how consistent the measurements would be if performed by different individuals on the same skull models. Additionally, all anatomical measurements in the study were obtained from 3D models reconstructed from CT images, without corresponding in vivo measurements and validation.

## Future outlook

Our study's measurement data may aid neurosurgeons in quickly and easily understanding the anatomical characteristics of the MMA and surrounding bony structures, which can help improve the preservation rate of the MMA to ensure patients achieve good outcomes. In the future, we hope to develop related surgical equipment that can allow us to use virtual reality technology to observe brain tissue structure in real-time during surgery, achieving the goal of intraoperative localization and guidance.

## Conclusion

The MMA plays a crucial role in cerebrovascular reconstruction surgery. Typically, the frontal branch's proximal portion of the MMA is obscured by a bony canal or deep groove in most patients. The distance between the end of this canal/groove and the midpoint of the pterion varies considerably, without a considerable difference between the left and right sides. However, the distance between the canal/groove's end and the upper edge of the zygomatic arch and the outer margin of the frontozygomatic suture exhibits less fluctuation. To prevent damage to the frontal branch of the MMA, creating a bone flap outside of the "Dangerous Rectangle" is an effective approach. Furthermore, incorporating a modified craniotomy procedure with simple VR technology based on 3D Slicer can optimize the preservation of the MMA's integrity. Consequently, this strategy is a cost-effective, efficient, and straightforward methodology.

## Data Availability

The data are available from the corresponding author on reasonable request approved the institutional review boards of Changshu First People's Hospital.

## Abbreviations

MMA: Middle meningeal artery
MMD: Moyamoya disease
ICAO: Internal carotid artery occlusion
VR: Virtual reality
3D: Three-dimensional
EDMS: Encephalo-duro-myo-synangiosis
EDAS: Encephalo-duro-arterio-synangiosis
EDAMS: Encephalo-duro-arterio-myosynangiosis
ACA: Anterior cerebral artery
MCA: Middle cerebral artery
DSA: Digital subtraction angiography
CTP: Computer tomography perfusion
MRP: Magnetic resonance perfusion
STA: Superficial temporal artery
NCCT: Non-contrast computer tomography
CT: Computer tomography
MR: Magnetic resonance
ICG: Indocyanine green
